# Effects of exogenous γ-aminobutyric acid concentrations on growth and nutrient uptake in trifoliate orange (*Poncirus trifoliata*) seedlings under boron-deficient and -sufficient conditions

**DOI:** 10.3389/fpls.2026.1839626

**Published:** 2026-07-23

**Authors:** Tian Jin, Yuemei Xu, Yawei Hu, Fengxian Yao, Guidong Liu

**Affiliations:** 1 College of Life Sciences, Gannan Normal University, Ganzhou, China; 2 National Navel Orange Engineering Research Center, Ganzhou, China

**Keywords:** abiotic stress, mineral homeostasis, plant growth regulators, root system, rootstock

## Abstract

Boron (B) deficiency is a major factor limiting plant growth and yield. Gamma-aminobutyric acid (GABA), a crucial non-protein amino acid in plants, plays vital roles in plant growth and stress responses. However, its specific regulatory role on nutrient homeostasis under B deficiency remains unclear. This study elucidated the regulatory effects of exogenous GABA on the growth and mineral element acquisition in trifoliate orange (*Poncirus trifoliata*) seedlings under both B-deficient and B-sufficient conditions. A completely randomized experiment with a two-way factorial design was conducted, where plants grown hydroponically were exposed to B-deficient or B-sufficient nutrient solutions, supplemented with five GABA concentrations (0.01, 0.05, 0.1, 0.5, and 1 mM). Our results showed that the effects of GABA are highly concentration-dependent. Under B deficiency, low concentrations of GABA significantly promoted plant growth, with the 0.1 mM treatment increasing plant biomass by 23.3% compared to the B-deficient control. These optimal treatments restored growth by improving root morphological traits and enhancing the accumulation of essential mineral elements (increasing specific nutrient accumulations by up to 27.6%). In contrast, high GABA concentrations exhibited toxic effects irrespective of B supply. Specifically, the 1 mM treatment decreased overall biomass by 8.9% and 19.2% under B-deficient and B-sufficient conditions, respectively. This study provides a physiological foundation for understanding GABA-mediated nutrient regulation, indicating its potential as a candidate for future nutrient management strategies under B-deficient conditions.

## 1 Introduction

Trifoliate orange (*Poncirus trifoliata*) is the most widely utilized deciduous rootstock in citrus production globally, particularly in China, owing to its excellent cold hardiness and disease resistance. Its robust root system largely determines the nutrient acquisition and overall vigor of the grafted scion (Liu et al., 2013). However, the productivity of trifoliate orange is frequently constrained by abiotic stresses, with boron (B) deficiency being a major global limitation (Yang et al., 2022; Chu et al., 2025).

Boron is an essential micronutrient critical for numerous physiological and biochemical processes, imparting significant benefits to agricultural production by maintaining the structural integrity required for cell elongation, primarily through the cross-linking of rhamnogalacturonan-II (RG-II) in cell walls (Voxeur and Fry, 2014; Eggert and von Wirén, 2017; Wang et al., 2021a). Consequently, trifoliate orange is highly susceptible to B deficiency (Liu et al., 2013; Zhou et al., 2014), which manifests as impaired root development, stunted growth, and specific leaf symptoms (e.g., chlorosis and corky split vein) (Han et al., 2008; Yang et al., 2022; Zhou et al., 2022). Although B fertilization is effective in rectifying deficiency symptoms, dosage control is extremely difficult in practice given that the optimal soil available B concentration for citrus resides within a narrow range (typically 0.5–1.0 mg kg^-^¹) (Yang et al., 2022). Continuous or improper B supplementation frequently results in B over-accumulation, severely compromising fruit yield and quality (Navarro-Pérez et al., 2024).

Boron deficiency directly impairs cell wall cross-linking, which frequently induces oxidative stress and disrupts cellular metabolic homeostasis (Wang et al., 2026). To withstand these stress-induced damages, a series of physiological responses are triggered. The activities of antioxidant enzymes, such as catalase and peroxidase, are significantly upregulated to mitigate excessive ROS accumulation (Li et al., 2023). Concurrently, phytohormone signaling networks are modulated to coordinate developmental adaptations; for instance, jasmonic acid and ethylene synergistically regulate primary root growth under B deficiency (Huang et al., 2021). Furthermore, the expression of specific aquaporin channels (e.g., NIP5;1) and B efflux transporters (e.g., BOR1) is significantly enhanced to maximize root B uptake and internal reallocation (Takano et al., 2006; Feng et al., 2020; Wang et al., 2021b). Despite these intrinsic biochemical and molecular responses, severe B deficiency ultimately leads to profound root growth inhibition and broad mineral nutrient imbalances, necessitating alternative external interventions to enhance plant resilience.

γ-aminobutyric acid (GABA), a ubiquitous four-carbon non-protein amino acid, has emerged as a crucial signaling molecule and regulator beyond its role in nitrogen metabolism (Gahlowt et al., 2024). In plants, GABA is synthesized via glutamate decarboxylation and degraded through the GABA shunt to enter the tricarboxylic acid (TCA) cycle, ensuring metabolic homeostasis (Hu et al., 2024). GABA accumulates rapidly in response to diverse abiotic stresses, including nutrient imbalances, to function in stress mitigation (Huang et al., 2023; Guo et al., 2023). For instance, under short-term NaCl and CdCl_2_ stress, elevated GABA levels regulate cellular osmotic potential and alleviate metabolic disorders (Ji et al., 2020). Furthermore, it modulates stomatal aperture via the anion transporter ALMT9, thereby impacting drought tolerance (Xu et al., 2021). Beyond stress responses, GABA plays significant regulatory roles in plant growth and development. In poplar and pine species, endogenous GABA modulates adventitious root formation and vascular development by interacting with hormone homeostasis and signal cascades (Xie et al., 2020). Importantly, GABA has been shown to inhibit root cell elongation by regulating root pH balance (Molina-Rueda et al., 2015).

Mineral nutrients are essential for plant growth and development, making the improvement of nutrient absorption and utilization efficiency crucial for enhancing plant resilience and sustainability (Wang et al., 2025). Recent studies indicate that GABA can regulate plant growth and development by influencing mineral element uptake. For example, exogenous GABA application increases nitrogen (N), phosphorus (P), and potassium (K) concentrations in loquat seedlings, enhancing their nutrient absorption capacity and promoting growth (Yang et al., 2023). Similarly, under conditions of excessive ammonium (NH_4_^+^), GABA alleviates rhizosphere acidification in rice, thereby promoting the uptake of calcium (Ca), magnesium (Mg), iron (Fe), and zinc (Zn) and mitigating ammonium toxicity (Ma et al., 2016). While GABA’s role in ameliorating abiotic stresses and enhancing the uptake of some nutrients is increasingly recognized, its specific functions in modulating plant responses to micronutrient deficiencies remain poorly explored (Jalil et al., 2025). Furthermore, GABA’s effects exhibit significant concentration dependence: beneficial impacts at lower concentrations can shift to inhibitory effects at higher concentrations. This duality is exemplified in Arabidopsis, where low GABA concentrations stimulate pollen tube elongation, while high concentrations inhibit it, demonstrating the essential role of GABA in guiding pollen tubes via concentration gradients (Palanivelu et al., 2003). This phenomenon, characterized by low-dose stimulation and high-dose inhibition, aligns with the concept of “hormesis”—a biphasic dose-response relationship that is increasingly recognized as a fundamental adaptive mechanism in plant responses to signaling molecules and environmental stressors (Calabrese and Blain, 2009).

Although GABA’s role in alleviating various nutrient stresses is recognized, its specific function under boron deficiency remains poorly understood. Therefore, this study aimed to elucidate the concentration-dependent regulatory effects of exogenous GABA on the growth and mineral element acquisition of trifoliate orange seedlings subjected to both B-deficient and B-sufficient conditions. We hypothesized that specific GABA concentrations would mitigate the detrimental effects of B deficiency not only by improving root morphological traits but also by rebalancing elemental concentrations across different plant tissues. Unlike previous studies that primarily investigated GABA’s protective roles under conventional abiotic stresses, this work comprehensively evaluated the interactive impacts of GABA and B on seedling biomass, root morphology, and the tissue-specific accumulation of key macro- and micronutrients. This approach broadens the understanding of GABA’s physiological functions, providing new insights into its potential as a modulator for nutrient management strategies in citrus orchards.

## 2 Materials and methods

### 2.1 Plant materials and treatment

Seeds of trifoliate orange (*Poncirus trifoliata*) were obtained from the nursery base of Gannan Normal University (Ganzhou, China). Seeds were surface-sterilized by immersion in 5% (v/v) sodium hypochlorite solution for 15 min, immediately followed by repeated rinsing with deionized water. Subsequently, seeds were treated with 75% (v/v) alcohol for 90 s and thoroughly rinsed five times with deionized water to ensure the complete removal of any residual sterilant. Sterilized seeds were sown on wet gauze and placed in an incubator at 28 °C to germinate. Germinated seeds were then transferred onto 0.6% (w/v) agar plates and maintained in a growth chamber under controlled conditions: light intensity of 400–440 μmol m^-2^ s ^-1^, photoperiod of 16 h light/8 h dark and temperature of 28 ± 2 °C (Yan et al., 2019).

When the seedlings grew to the four true-leaf stage, uniform seedlings were selected and transplanted into 12 × 8 × 11 cm (length × width × height) plastic containers filled with 1 L of nutrient solution. Each container contained a modified Hoagland’s No.2 solution (Hoagland and Arnon, 1950). The solution composition was: 1.5 mM KNO_3_, 1 mM Ca(NO_3_)_2_, 0.5 mM MgSO_4_, 0.14 mM Na_2_HPO_4_, 0.32 mM NaH_2_PO_4_, 4.5 μM MnCl_2_, 0.8 μM ZnSO_4_, 0.15 μM CuSO_4_, 0.1 μM Na_2_MoO_4_, 25 μM iron-ethylenediaminetetraacetic acid (EDTA-Fe), and 10 μM H_3_BO_3_ (with continuous aeration for 20 min every 2 h and renewal every 3 days).

Seedlings were initially pre-cultured in 1 L plastic containers filled with full nutrient solution for 15 days to ensure uniform growth. Following this period, the nutrient solution in each container was replaced with the respective treatment solutions to initiate the experiment. Treatments combined two B levels (B sufficiency, **+**B, 10 μM H_3_BO_3_; B deficiency, **-**B, 0 μM H_3_BO_3_) with six concentrations of GABA: 0 (0G), 0.01 (0.01G), 0.05 (0.05G), 0.1 (0.1G), 0.5 (0.5G), and 1 (1G) mM GABA. For these treatments, GABA was added to the respective nutrient solutions using a freshly prepared 1 M stock solution. After the complete addition of all nutrient components and GABA, the pH of the final solutions was uniformly adjusted to 5.8 using 0.5 M H_2_SO_4_ and 1 M NaOH. Each treatment combination was replicated in three plastic containers, with five seedlings per container.

The experiment was laid out in a completely randomized design with a two-way factorial arrangement. Each hydroponic container, rather than the individual seedling, was designated as a single independent biological replicate (*n* = 3 containers per treatment). Each container contained five seedlings, giving a total of 15 seedlings per treatment. The seedlings were grown under these respective treatment conditions for 30 days before harvesting for subsequent morphological and physiological analyses.

### 2.2 Determination of plant growth parameters

To evaluate the effects of GABA and boron treatments on plant growth, several growth parameters were determined, including stem length, total root length, primary root length, root surface area, root volume, total fresh weight, and total dry weight. For growth analysis, all five seedlings within each container were harvested, and their measurements were averaged to represent that container. Stem length and primary root length were measured by a ruler. The morphology of the whole plant was photographed using a digital camera. Root morphological parameters (e.g. total root length, surface area, volume, diameter) were analyzed using an Epson scanner (Expression 10000XL 1.0) and WinRhizo software (Pro v. 2009c). A subset of roots (~1 cm sections from the tips of representative samples) was excised for examination under a stereoscopic microscope. Seedlings designated for biomass determination were washed thoroughly with ultrapure water to remove any nutrient solution residues. Surface moisture was gently blotted using absorbent paper, and fresh weight was recorded immediately for the whole seedling. Subsequently, the seedlings were separated into roots, stems, and leaves. All plant tissues were then oven-dried at 70 °C for 72 h until constant weight was achieved to determine dry weight. Constant weight was defined as a weight change of less than 0.2% between two consecutive weighing at a 24-h interval.

### 2.3 Determination of mineral nutrients

For determination of mineral nutrients, dried plant tissues from the five seedlings within each container were physically pooled and homogenized. From this pooled sample, 0.10 g aliquots of dried plant tissue were subjected to dry ashing. Samples were ashed in a muffle furnace at 550 °C for 6 h. The cooled ash residues were dissolved in 5 mL of 5% (v/v) nitric acid (HNO_3_). Mineral nutrient concentrations (including B, P, K, Ca, Mg, Fe, Mn, Cu, Zn and Mo) in these acid digests were quantified using inductively coupled plasma mass spectrometry (ICP-MS; Agilent 7900, USA) (Liu et al., 2020). Separately for total nitrogen quantification, ~0.10 g dry samples were digested with concentrated sulfuric acid-hydrogen peroxide (H_2_SO_4_-H_2_O_2_) mixture. The resulting digestate was analyzed for total nitrogen concentration via automated colorimetric assay using a discrete chemical analyzer (SmartChem 200, AMS Alliance, France) (Zhang et al., 2023).

### 2.4 Statistical analysis

Statistical analyses were performed using SPSS Statistics 22.0 (IBM Corp., Armonk, NY, USA). Prior to ANOVA, the homogeneity of variances was verified using Levene’s test. A two-way analysis of variance (ANOVA) was conducted to evaluate the main effects of B levels, GABA concentrations, and their interactions. To elucidate the specific differences among all experimental conditions, *post hoc* multiple comparisons were performed across all twelve treatment combinations using Duncan’s multiple range test (*P* < 0.05). Accordingly, different lowercase letters in the figures indicate significant differences among all 12 treatments. In the figures, significance levels from the two-way ANOVA are denoted as follows: *P < 0.05, **P < 0.01, and ***P < 0.001. All data are presented as mean ± standard deviation (SD). To explore the relationships among samples based on mineral nutrient concentrations, principal component analysis (PCA) was performed on standardized data (Z-score normalization) to account for differences in units and scales across elements. The PCA was conducted using OriginPro software (version 2023b; OriginLab, Northampton, MA, USA), and the results are presented as score plots and loading plots.

## 3 Results

### 3.1 Plant growth and biomass

**Figure 1:**
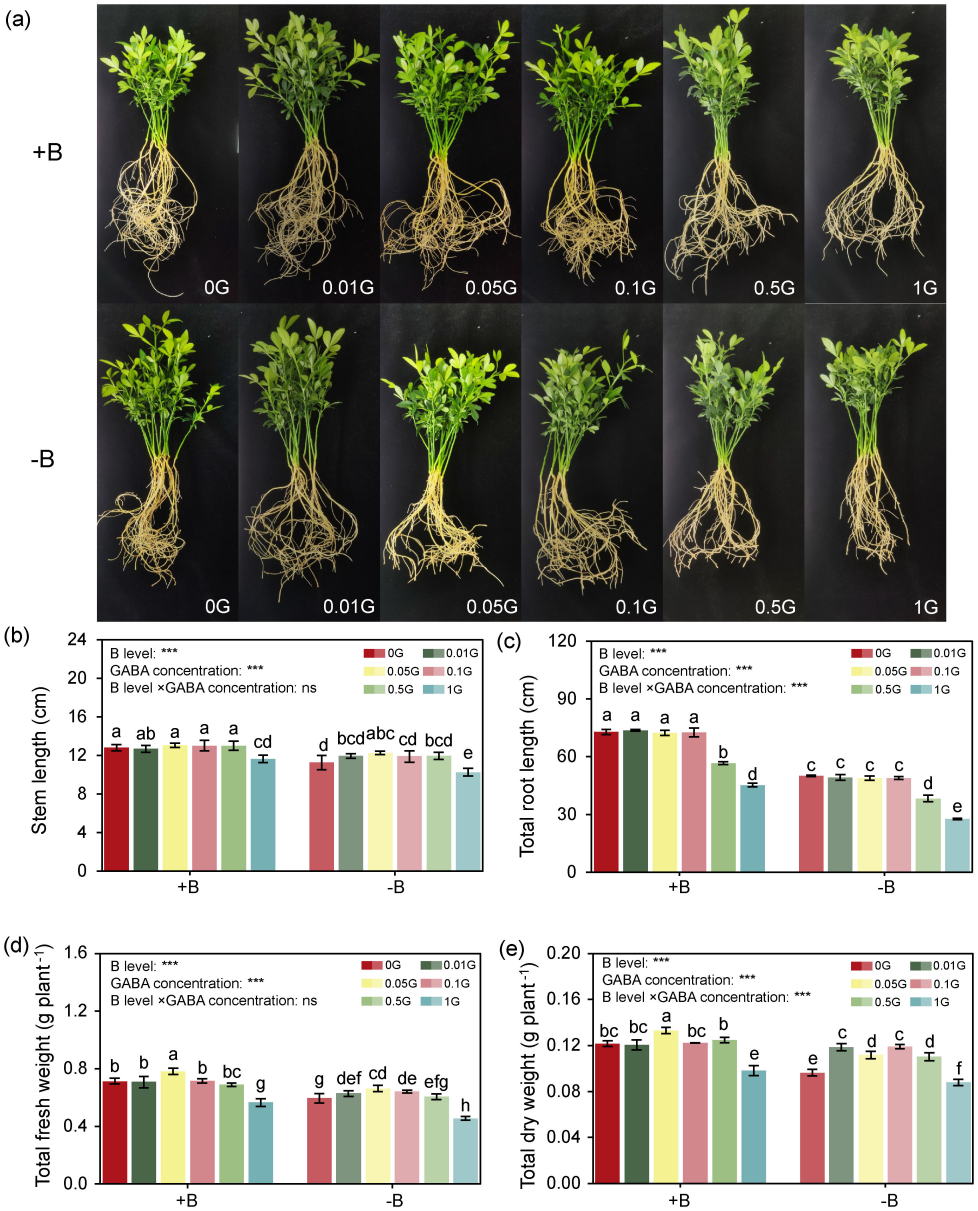
Interactive effects of exogenous GABA and boron (B) supply on the biomass and growth parameters of trifoliate orange seedlings. **(a)** Morphology of seedlings; **(b)** stem length; **(c)** total root length; **(d)** total fresh weight; and **(e)** total dry weight. Seedlings were subjected to B-sufficient (+B) and B-deficient (-B) conditions. 0G, 0.01G, 0.05G, 0.1G, 0.5G, and 1G represent GABA concentrations of 0, 0.01, 0.05, 0.1, 0.5, and 1 mM, respectively. Two-way ANOVA was used to evaluate the effects of B level, GABA concentration, and their interaction. Asterisks denote significance at the *P < 0.05, **P < 0.01, and ***P < 0.001 levels. Different lowercase letters indicate significant differences (*P* < 0.05) among all 12 treatment combinations (comparing both B levels and all GABA concentrations simultaneously) according to Duncan’s multiple range test. Error bars represent SD of three biological replicates (containers).

Two-way ANOVA results showed that both B level and GABA concentration exerted significant effects on stem length, total root length, total fresh weight, and total dry weight. Moreover, a significant interaction between B level and GABA concentration was observed for total root length and total dry weight (**Figure 1**). Compared to B-sufficient controls, B-deficient seedlings exhibited significantly lower biomass and growth traits (*P* < 0.05), with stem length, total root length, total fresh weight, and total dry weight decreasing by 12.0%, 31.3%, 16.5%, and 20.7%, respectively. In the B-deficient seedlings, the stem length and total fresh weight increased at GABA concentrations ≤ 0.05 mM but decreased when GABA exceeded 0.05 mM. Notably, at 1 mM GABA, total root length and dry weight were also significantly decreased compared to the B deficiency without GABA treatment.

Under B-sufficient conditions, GABA concentrations of 0.01–0.1 mM elicited no significant changes in stem or total root length. However, the biomass increased with 0.05 mM GABA compared to the control. Higher concentrations (0.5 and 1 mM GABA) reduced total root length. At 1 mM GABA, the stem length, total root length, total fresh weight, and total dry weight were significantly reduced by 8.5%, 37.9%, 20.7%, and 19.2%, respectively (*P* < 0.05).

### 3.2 Root morphology

**Figure 2:**
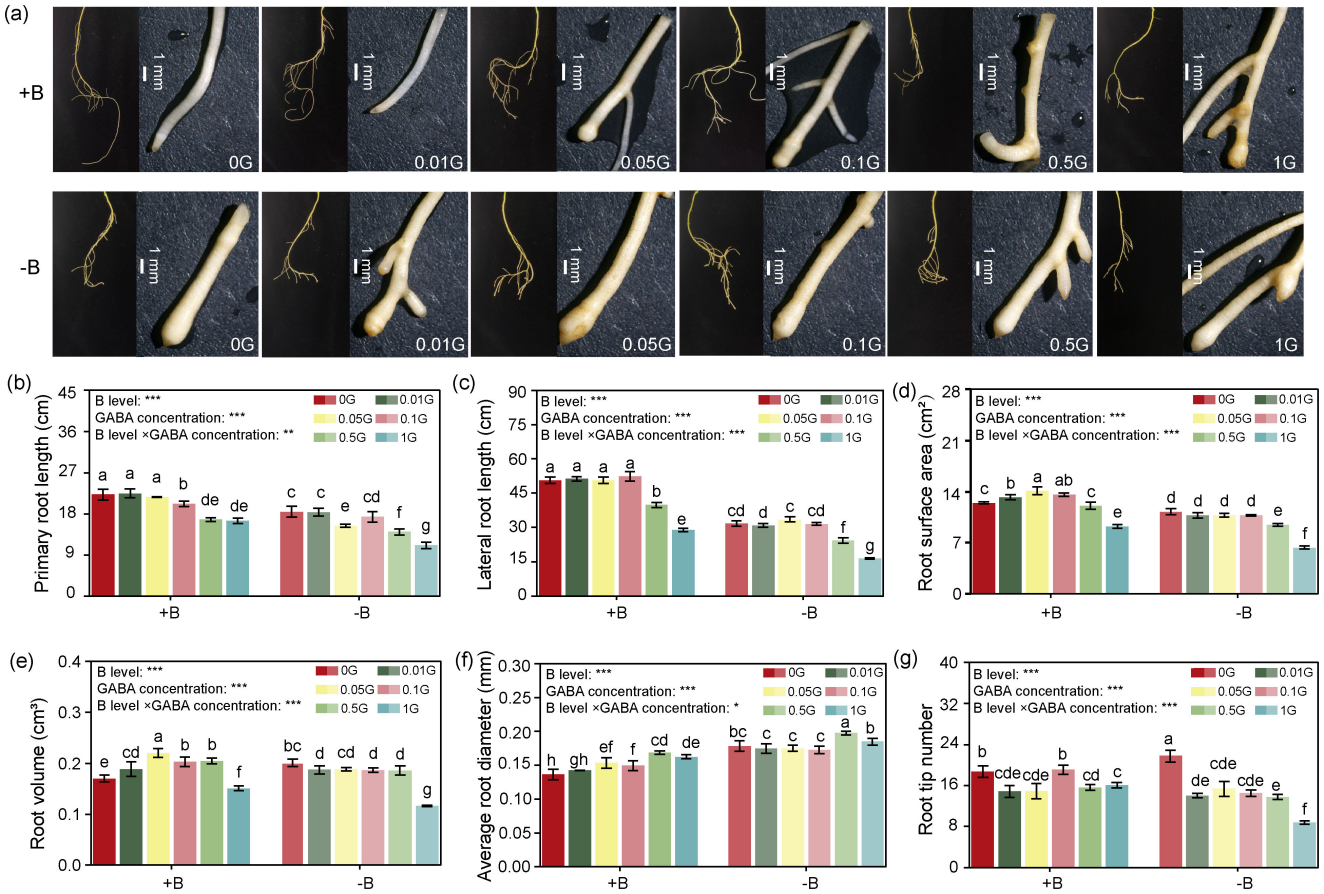
Interactive effects of exogenous GABA and boron (B) supply on root morphological parameters of trifoliate orange seedlings. **(a)** Morphology of a single plant and root tip; **(b)** primary root length; **(c)** lateral root length; **(d)** root surface area; **(e)** root volume; **(f)** average root diameter; and **(g)** root tip number. Seedlings were grown under B-sufficient (+B) and B-deficient (-B) conditions. 0G, 0.01G, 0.05G, 0.1G, 0.5G, and 1G represent GABA concentrations of 0, 0.01, 0.05, 0.1, 0.5, and 1 mM, respectively. Two-way ANOVA was used to evaluate the effects of B level, GABA concentration, and their interaction. Asterisks denote significance at the *P < 0.05, **P < 0.01, and ***P < 0.001 levels. Different lowercase letters indicate significant differences (*P* < 0.05) among all 12 treatment combinations (comparing both B levels and all GABA concentrations simultaneously) according to Duncan’s multiple range test. Error bars represent SD of three biological replicates (containers).

Root morphological parameters were significantly affected by B levels, GABA concentration, and their interaction (**Figures 2b–g**). Compared to the control plants, the B-deficient seedlings exhibited swollen root tips and increased lateral root emergence predominantly in basal root regions (**Figure 2a**). Exogenous GABA application induced root tip thickening in both B-deficient and B-sufficient seedlings, while promoting lateral root formation near root apices. Notably, high concentrations of GABA even caused primary root tip necrosis.

The primary root length, lateral root length, and root surface area of the B-deficient seedlings were reduced by 16.8%, 20.7%, and 9.8%, respectively, compared to those of the control (**Figures 2b–d**), while the root volume, average root diameter, and root tip number increased by 18.2%, 30.8%, and 16.5%, respectively (**Figures 2e–g**). Under B-deficient conditions, exogenous GABA application significantly reduced the number of root tips relative to untreated B-deficient plants. GABA concentrations of 0.5 and 1 mM further decreased primary root length, lateral root length, and root surface area. In B-sufficient seedlings, root surface area and volume showed a dose-dependent response: both parameters increased at GABA concentrations ≤ 0.05 mM but decreased at higher concentrations. Conversely, all tested GABA concentrations (0.01–1 mM) increased average root diameter. Notably, 1 mM GABA treatments consistently reduced root surface area and volume.

### 3.3 Mineral element concentrations in roots

**Figure 3:**
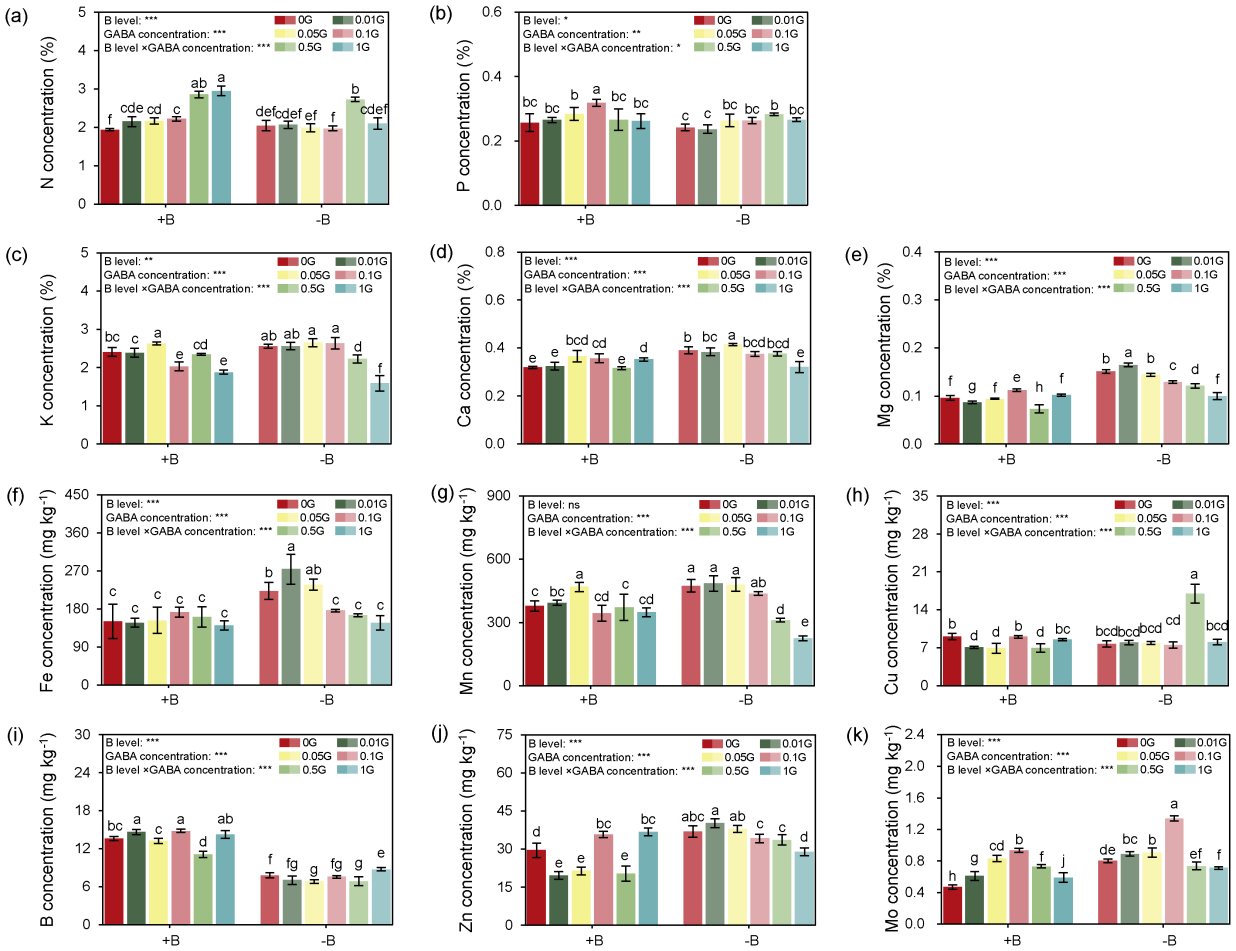
Interactive effects of exogenous GABA and boron (B) supply on the concentrations of mineral elements including N **(a)**, P **(b)**, K **(c)**, Ca **(d)**, Mg **(e)**, Fe **(f)**, Mn **(g)**, Cu **(h)**, B **(i)**, Zn **(j)**, and Mo **(k)** in roots of trifoliate orange seedlings. Seedlings were subjected to B-sufficient (+B) and B-deficient (-B) conditions. 0G, 0.01G, 0.05G, 0.1G, 0.5G, and 1G represent GABA concentrations of 0, 0.01, 0.05, 0.1, 0.5, and 1 mM, respectively. Two-way ANOVA was used to evaluate the effects of B level, GABA concentration, and their interaction. Asterisks denote significance at the *P < 0.05, **P < 0.01, and ***P < 0.001 levels. Different lowercase letters indicate significant differences (*P* < 0.05) among all 12 treatment combinations (comparing both B levels and all GABA concentrations simultaneously) according to Duncan’s multiple range test. Error bars represent SD of three biological replicates (containers).

Root B concentration decreased significantly in B-deficient seedlings compared to B-sufficient controls, while the concentrations of Ca, Mg, Fe, Mn, Zn, and Mo increased (**Figure 3**). Under B deficiency, GABA application induced a biphasic concentration response: Ca, Mg, Fe, and Mo initially increased then decreased relative to untreated B-deficient seedlings with rising GABA concentrations. At 1 mM GABA, the concentrations of K, Ca, Mg, Fe, Mn, Zn, and Mo decreased significantly in the B-deficient group.

Under B-sufficient conditions, GABA application significantly increased root N concentrations across all tested concentrations. Phosphorus and Mo concentrations increased at GABA concentrations ≤ 0.1 mM but decreased at higher concentrations. Similarly, K and Mn exhibited a hormetic response: concentrations peaked at 0.05 mM GABA then declined with further concentration increases. When the GABA concentration was higher than 0.5 mM, most mineral element concentrations in the roots showed a decreasing trend.

### 3.4 Mineral element concentrations in stem

**Figure 4:**
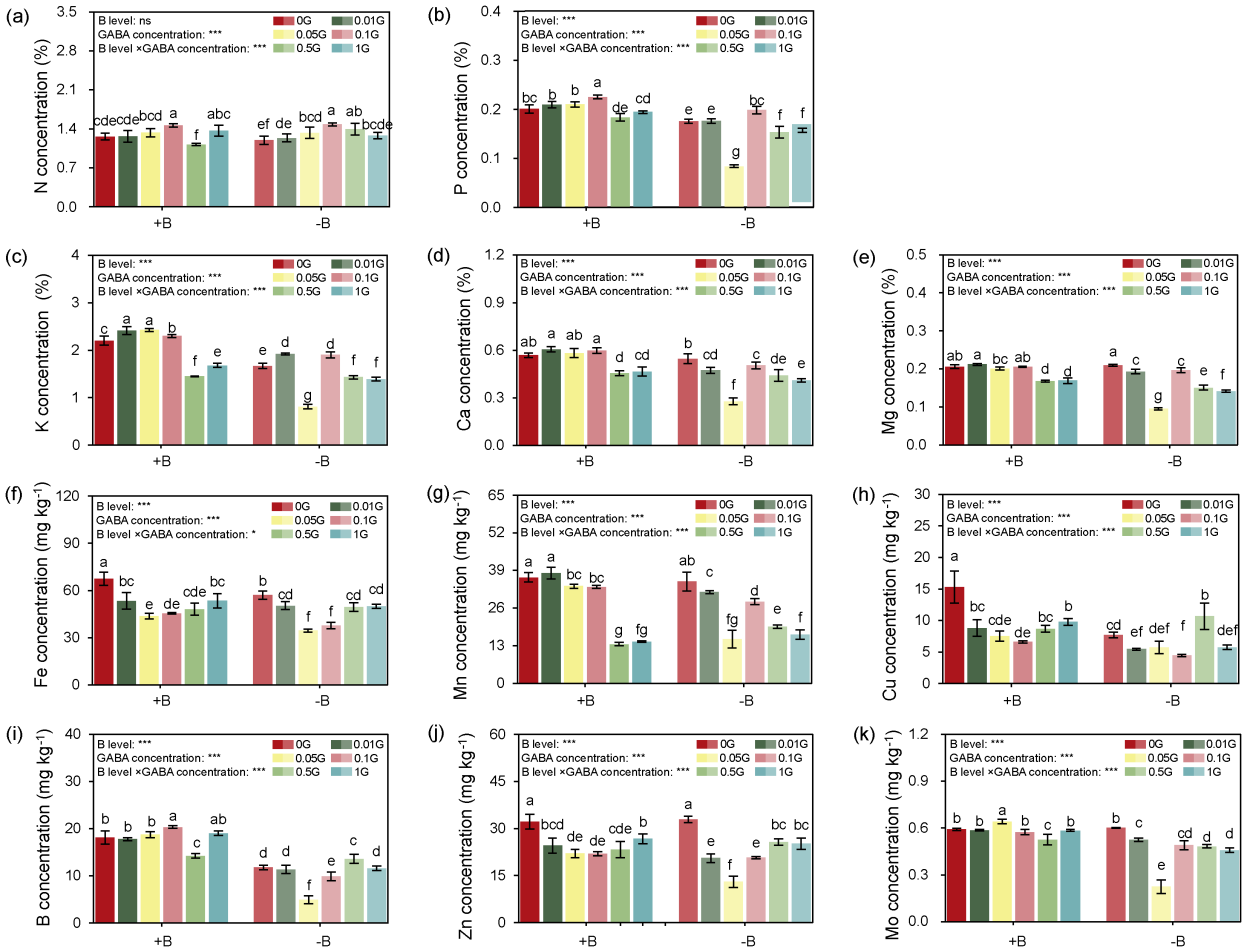
Interactive effects of exogenous GABA and boron (B) supply on the concentrations of mineral elements including N **(a)**, P **(b)**, K **(c)**, Ca **(d)**, Mg **(e)**, Fe **(f)**, Mn **(g)**, Cu **(h)**, B **(i)**, Zn **(j)**, and Mo **(k)** in stems of trifoliate orange seedlings. Seedlings were subjected to B-sufficient (+B) and B-deficient (-B) conditions. 0G, 0.01G, 0.05G, 0.1G, 0.5G, and 1G represent GABA concentrations of 0, 0.01, 0.05, 0.1, 0.5, and 1 mM, respectively. Two-way ANOVA was used to evaluate the effects of B level, GABA concentration, and their interaction. Asterisks denote significance at the *P < 0.05, **P < 0.01, and ***P < 0.001 levels. Different lowercase letters indicate significant differences (P < 0.05) among all 12 treatment combinations (comparing both B levels and all GABA concentrations simultaneously) according to Duncan’s multiple range test. Error bars represent SD of three biological replicates (containers).

Compared with the control, the concentrations of P, K, Fe, Cu, and B in the stems of B-deficient seedlings were significantly reduced, while the concentrations of other mineral elements showed no significant differences (**Figure 4**). With increasing GABA concentration, the N concentration in the stems of B-deficient seedling first increased, peaking at the 0.1 mM GABA treatment, and then decreased. Compared with the B-deficient treatment, the concentrations of P, K, Ca, Mg, Fe, Mn, B, Zn, and Mo decreased drastically under the 0.05 mM GABA treatment, followed by an increase with higher GABA concentrations. Except for N, K, Cu, and B, the concentrations of other mineral elements were significantly lower under the 0.1, 0.5 and 1 mM GABA treatments than under the B-deficient treatment.

Under B-sufficient conditions, stem Fe, Cu, and Zn concentrations exhibited biphasic responses to GABA application, decreasing initially then increasing with rising concentrations. At the 0.01 mM GABA treatment, Fe showed the most pronounced decrease among these elements. The 0.1 mM GABA significantly reduced stem Cu and Zn concentrations relative to the B-sufficient controls. Higher GABA concentrations (0.5 and 1 mM) decreased K, Ca, Mg, Fe, Mn, Cu, and Zn concentrations. Notably, Mn demonstrated exceptional sensitivity to elevated GABA, decreasing by 63.0% (0.5 mM) and 60.5% (1 mM) compared to the controls.

### 3.5 Mineral element concentrations in leaves

**Figure 5:**
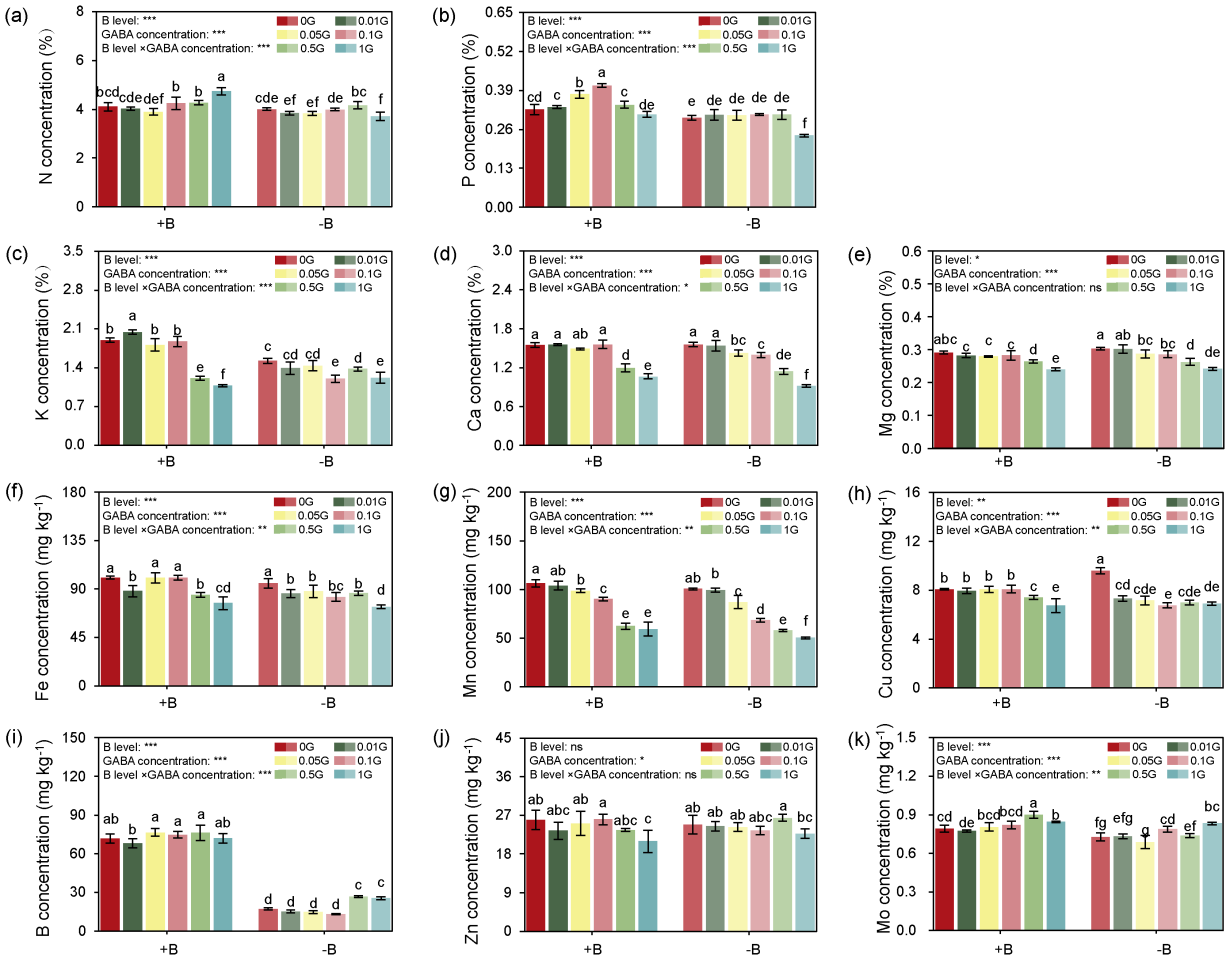
Interactive effects of exogenous GABA and boron (B) supply on the concentrations of mineral elements including N **(a)**, P **(b)**, K **(c)**, Ca **(d)**, Mg **(e)**, Fe **(f)**, Mn **(g)**, Cu **(h)**, B **(i)**, Zn **(j)**, and Mo **(k)** in leaves of trifoliate orange seedlings. Seedlings were subjected to B-sufficient (+B) and B-deficient (-B) conditions. 0G, 0.01G, 0.05G, 0.1G, 0.5G, and 1G represent GABA concentrations of 0, 0.01, 0.05, 0.1, 0.5, and 1 mM, respectively. Two-way ANOVA was used to evaluate the effects of B level, GABA concentration, and their interaction. Asterisks denote significance at the *P < 0.05, **P < 0.01, and ***P < 0.001 levels. Different lowercase letters indicate significant differences (*P* < 0.05) among all 12 treatment combinations (comparing both B levels and all GABA concentrations simultaneously) according to Duncan’s multiple range test. Error bars represent SD of three biological replicates (containers).

Boron deficiency significantly reduced leaf P, K, B, and Mo concentrations, while increasing Cu levels relative to B-sufficient controls (**Figure 5**). GABA application decreased Mg, Fe, Mn, and Cu concentrations in the B-deficient leaves. Magnesium and Mn levels exhibited progressive decreases with increasing GABA concentrations. Concentrations of K and Ca declined specifically at 0.5 and 1 mM GABA. In contrast, these higher GABA concentrations (0.5 and 1 mM) elevated B concentrations by 54.1% and 47.1% relative to untreated B-deficient seedlings, respectively.

Under B-sufficient conditions, low GABA concentrations had no significant effect on most leaf mineral element concentrations, whereas higher concentrations (0.5 and 1 mM) significantly reduced most element levels. Specifically, K, Ca, Mg, Cu, Zn, and Fe concentrations decreased significantly following treatment with 0.5 and 1 mM GABA. The concentrations of P also stopped increasing and decreased when GABA exceeded 0.1 mM. In contrast, N and Mo concentrations exhibited progressive increases across the tested GABA concentration gradient.

### 3.6 Mineral element accumulation and allocation in seedlings

**Figure 6:**
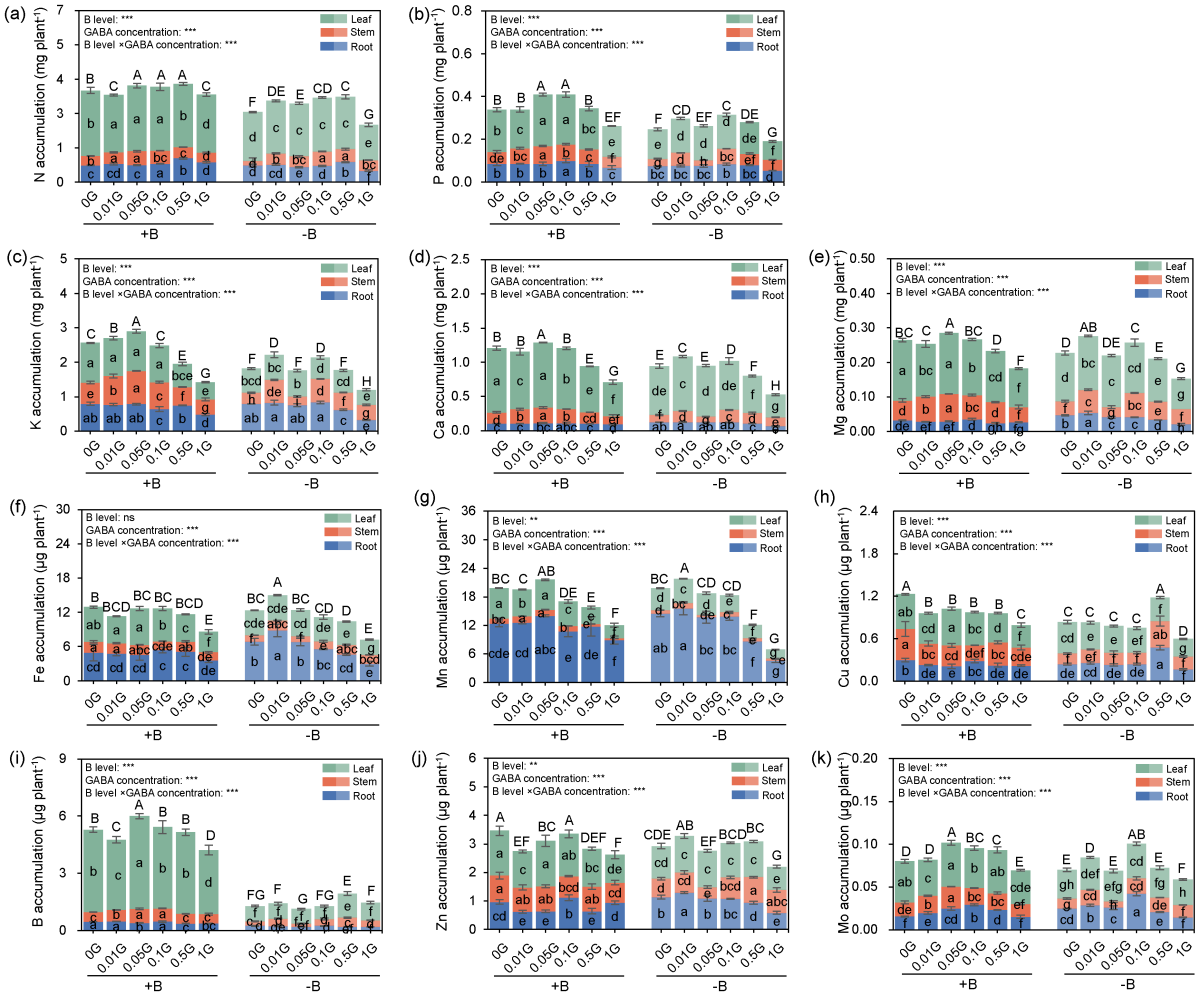
Interactive effects of exogenous GABA and boron (B) supply on the accumulation and allocation of mineral elements including N **(a)**, P **(b)**, K **(c)**, Ca **(d)**, Mg **(e)**, Fe **(f)**, Mn **(g)**, Cu **(h)**, B **(i)**, Zn **(j)**, and Mo **(k)** in trifoliate orange seedlings. Seedlings were subjected to B-sufficient (+B) and B-deficient (-B) conditions. 0G, 0.01G, 0.05G, 0.1G, 0.5G, and 1G represent GABA concentrations of 0, 0.01, 0.05, 0.1, 0.5, and 1 mM, respectively. Two-way ANOVA was used to evaluate the effects of B level, GABA concentration, and their interaction. Asterisks denote significance at the *P < 0.05, **P < 0.01, and ***P < 0.001 levels. According to Duncan’s multiple range test, different lowercase letters indicate significant differences (*P* < 0.05) in mineral accumulation within specific tissues (roots, stems, or leaves) across all 12 treatment combinations, while different uppercase letters indicate significant differences (*P* < 0.05) in the total accumulation across all 12 treatment combinations. Error bars represent SD of three biological replicates (containers).

The accumulation of mineral elements in the whole seedlings was significantly influenced by B level, GABA concentrations, and their interaction, except for Fe accumulation which remained unaffected by B levels (**Figure 6**). Under B deficiency, nutrient accumulation (N, P, K, Ca, Mg, Cu, B, Zn, and Mo) was significantly inhibited by a preferential allocation to roots. Exogenous GABA partially mitigated these deficiency effects in a concentration-dependent manner: low concentrations (0.01–0.1 mM) generally improved the accumulation of macronutrients (N, P, K, Ca, and Mg), whereas the highest concentration (1 mM) showed a significant inhibitory effect on all elements except B. Furthermore, GABA application redirected mineral partitioning; for instance, low doses (0.01–0.05 mM) favored the mobilization of nutrients toward stems and leaves, effectively counteracting the B-deficiency-induced root sequestration.

Under B-sufficient conditions, 0.05 mM GABA significantly improved the N, P, K, Ca, Mg, B, and Mo accumulation in seedlings compared to the untreated control. Conversely, 1 mM GABA suppressed accumulation of all measured mineral elements except Mo. GABA at 0.01–0.1 mM decreased root K allocation ratios, whereas 0.5–1 mM GABA increased them. In contrast, the allocation of B to the roots increased by 0.01, 0.1, and 0.5 mM GABA, but decreased at 0.05 and 1 mM GABA. Across all GABA concentrations, root allocation ratios increased for N, P, Ca, Mg, Fe, and Mn. Conversely, stem allocation increased for P, K, Ca, and Mg but impeded for Fe, Mn, and Cu. Leaf allocation ratios universally decreased for N, P, K, Ca, Mg, Mn, B, and Zn.

### 3.7 PCA of mineral elements in the roots, stems, and leaves of trifoliate orange seedlings

**Figure 7:**
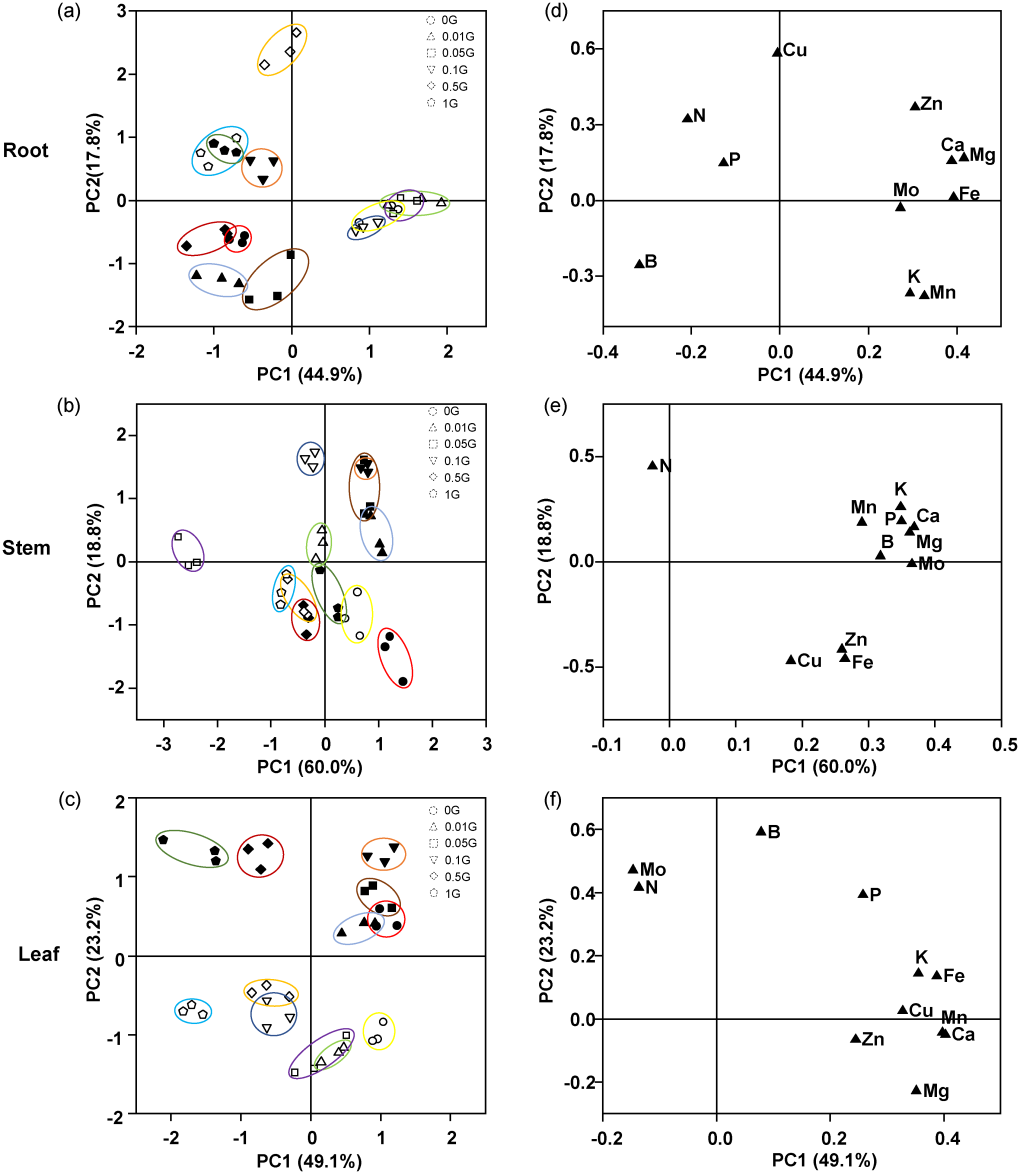
PCA scoring plots **(a–c)** and loading plots **(d–f)** of the mineral element concentrations with different treatments. Solid and open symbols represent boron-sufficient and boron-deficient conditions, respectively.

PCA of standardized mineral profiles revealed tissue-specific treatment separations (**Figure 7**). In roots (62.7% total variance), B-deficient and B-sufficient groups showed distinct segregation, except for the 1 mM GABA treatment under B deficiency. B-deficient seedlings with 0–0.1 mM GABA clustered along PC1 (44.9%, driven primarily by Mg, Fe, and Ca), while PC2 (17.8%) separated B-sufficient seedlings with 0.05 mM and B-deficient seedlings with 0.5 mM GABA treatments (**Figure 7a**). In stems (78.8% variance), PC1 (60.0%, driven by Ca, Mg, and Mo) distinguished the 0.05 mM GABA-treated B-deficient seedlings from the B-deficient control (0 mM GABA), and separated the 0.01–0.1 mM GABA treatments under B-sufficient condition from the 0.5 mM GABA treatment under B-sufficient condition; PC2 (18.8%) separated B-deficient seedlings treated with 0.5 mM GABA from the B-deficient control (**Figure 7b**), and distinguished all GABA-treated B-sufficient seedlings from the B-sufficient control. Leaf PCA (72.3% variance) exhibited pronounced B-deficiency/B-sufficiency separation, where PC1 (49.1%) isolated the highest GABA levels (0.5 and 1 mM) from other concentrations within each B-status group (**Figure 7c**). Concurrently, PC2 (23.2%) separated the B-deficient seedlings treated with 0.1–1 mM GABA from the other B-deficient seedlings.

## 4 Discussion

This study demonstrates that exogenous GABA exerts concentration-dependent effects on growth and nutrient homeostasis in trifoliate orange seedlings, with outcomes critically modulated by B availability. While B deficiency predictably suppressed growth and disrupted mineral accumulation, GABA application produced divergent responses contingent upon both concentration and B status, thereby confirming our primary hypothesis regarding context-dependent GABA efficacy. Specifically, the observed response curves for biomass and nutrient accumulation across the GABA gradient are consistent with typical hormetic behavior, where optimal physiological benefits are achieved at low doses.

Consistent with reports that B deficiency causes defective root morphology (Brdar-Jokanovic, 2020), our study demonstrated that B-deficient trifoliate orange seedlings suffered significant reductions in growth traits, accompanied by root tip swelling. In alignment with previous studies on GABA’s stress-alleviating roles (Shi et al., 2023; Lin et al., 2024), our study revealed that low GABA concentrations (0.01–0.1 mM) restored biomass in B-deficient seedlings. Under B-sufficient conditions, the highest values for seedling biomass, root volume, and surface area were specifically observed at 0.05 mM GABA, evidencing promoted root thickening. We interpret this phenomenon as an adaptive structural reconfiguration, further supported by our observation that GABA induced lateral root formation at root tips irrespective of B status. We hypothesize that such morphological shifts might involve GABA-mediated auxin signaling. Although endogenous auxin concentrations were not directly quantified in the present study, analogous developmental patterns have been reported in other species (Boerjan et al., 1995; Guo et al., 2020), thereby presenting a theoretical framework for future mechanistic investigations. Conversely, elevated GABA concentrations (≥ 0.5 mM) failed to mitigate B deficiency and caused root tip necrosis, further inhibiting seedling growth. The lowest values of these growth parameters observed at 1 mM GABA represent a severe phytotoxic phenotype. In this context, the root thickening under high GABA reflects a stress-induced morphogenic response (SIMR) rather than adaptive growth (Potters et al., 2007), which may be caused by excessive GABA impairing longitudinal cell elongation through cell wall-related processes (Renault et al., 2011). Moreover, our finding that root development was significantly inhibited at 0.5 mM GABA, whereas stem length reached its minimum only at 1 mM GABA indicates that roots exhibit greater GABA sensitivity than aerial tissues.

Boron deficiency significantly impedes the accumulation of essential macronutrients (N, P, K, Ca, Mg) and specific micronutrients (Zn, Mo, B) in trifoliate orange seedlings, disrupting critical physiological processes, consistent with the findings of Chanling et al. (2025). Critically, exogenous optimal GABA application counteracted these inhibitory effects and promoted nutrient accumulation under B-sufficient conditions, revealing its regulatory role in plant mineral nutrition (Yang et al., 2023; Niharika et al., 2025). These regulatory effects appeared to be primarily mediated by a strategic reconfiguration of root architecture. While B deficiency caused typical root tip swelling and reduced root surface area, low GABA concentrations induced a distinct morphological shift by promoting lateral root formation specifically near the root apices, thereby reducing the excessive root tips associated with B-deficiency. This specific structural adjustment may have contributed to optimizing the functional absorptive zones of the seedlings. A comparable phenomenon was observed in apple seedlings, where GABA-activated auxin signaling reoriented root development to facilitate phosphorus uptake (Chen et al., 2023). Consequently, low GABA concentrations (0.01–0.1 mM) significantly attenuated B deficiency-induced reductions in macronutrient uptake and enhanced the relocation of Ca and Mg to aerial parts, promoting photosynthesis and mitigating stress (Wang et al., 2020; Karabourniotis et al., 2020). Such physiological improvements parallel the findings of Cruz-Álvarez et al. (2024), who reported that foliar B application enhanced antioxidant enzyme activities (e.g., SOD and GPx) and optimized leaf N, K, and Ca concentrations, thereby reducing oxidative stress. Similarly, these low GABA concentrations promoted the uptake of micronutrients, notably Fe, Mn, Zn, and Mo, which may be linked to putative changes in transporter activities (Riaz and Guerinot, 2021; Saboor et al., 2021) and potential influences on rhizosphere pH via the downregulation of ALMT-mediated malate efflux (Bown and Shelp, 2020; Xu et al., 2021; Morton, 2023).

However, the regulation of ion homeostasis by GABA exhibited pronounced concentration dependence. While GABA acts as a nitrogen source, enhancing N levels which in turn promotes P and K absorption synergistically (Zhang et al., 2021), concentrations exceeding 0.1 mM (0.5 and 1 mM) reversed these benefits. The reduction in nutrient accumulation at these higher concentrations indicates a disruption of ion homeostasis. High GABA inhibited P and K accumulation, likely by inducing luxury N consumption, and reduced Cu and Mn accumulation, potentially due to antagonistic interactions with the promoted Mo uptake (Xu et al., 2021; Yang et al., 2021; Long and Peng, 2023). PCA results further corroborated this, clearly separating the beneficial low-dose effects (0.01–0.1 mM) from the inhibitory high-dose profiles (0.5–1 mM) based on distinct tissue-specific mineral signatures. Importantly, the major loading variables driving this segregation (primarily Ca, Mg, and Fe) highlight GABA’s specific role in nutrient rebalancing. Boron deficiency typically induces secondary ionic disorders. The statistical prominence of these elements indicates that GABA mitigates these disorders primarily by restoring the homeostasis of essential divalent cations and transition metals. Notably, the distinct elemental partitioning observed suggests that high GABA concentrations might have primarily impaired nutrient transport from roots to shoots rather than solely affecting root assimilation capacity (Zhu et al., 2021). Furthermore, particularly under B-deficient conditions, the 1 mM GABA treatment significantly reduced seedling biomass but increased leaf B concentration. This increase is likely not due to enhanced B uptake. Instead, it can be explained by a “concentration effect” resulting from the severe reduction in plant growth (Jarrell and Beverly, 1981; Gao et al., 2024). Since the high GABA concentration exerted a toxic effect that inhibited biomass accumulation, the passively absorbed B was concentrated within a much smaller tissue volume. Therefore, the altered nutrient distribution observed at 1 mM GABA reflects a disruption between plant growth and nutrient accumulation, rather than an active regulatory mechanism.

Overall, this biphasic dose response aligns with the principle of hormesis (Calabrese, 2013), indicating that GABA functions as a concentration-dependent signaling molecule rather than solely as a stress mitigator. Low concentrations of GABA exert beneficial effects, which are consistent with the established role of GABA in enhancing antioxidant capacity and interacting with stress signaling networks (Kumari et al., 2024). In contrast, excessive concentrations overwhelm cellular regulatory mechanisms, thereby disrupting metabolic homeostasis (Kathiresan et al., 1997; Renault et al., 2011). We hypothesize that this toxicity leads to an energy drain, which theoretically could involve feedback inhibition of key enzymes, disturbances in cytosolic pH buffering, or unintended activation of downstream signaling cascades (Palanivelu et al., 2003; Sze and Chanroj, 2018). Furthermore, a key question arising from this study is the extent to which the observed GABA effects are specific to B deficiency versus representing a general response to nutrient limitation. To achieve a deeper mechanistic understanding, future research must determine whether GABA preferentially modulates pathways directly associated with B sensing or common signaling hubs activated during nutrient stress. Potential cross-talk points include calcium signaling, reactive oxygen species dynamics, and specific hormone pathways (such as auxin) (Jia et al., 2022). To comprehensively test these hypotheses and overcome the limitations of our single-harvest (30 days) observation under controlled hydroponic conditions with relatively limited biological replication, future investigations should prioritize quantifying endogenous GABA accumulation, monitoring root-zone pH changes, and employing time-course experiments to capture the temporal dynamics of GABA-mediated regulation.

This study provides a physiological proof of concept that optimal exogenous GABA regulates nutrient uptake, promotes growth, and significantly enhances tolerance to B deficiency. Crucially, GABA acts as a physiological modulator that enhances plant resilience and the efficient utilization of existing internal nutrients; it does not serve as a chemical substitute for B. These findings provide a theoretical foundation for optimizing nutrient management strategies, particularly in soils prone to B deficiency or where plants exhibit delayed responses to direct B supplementation. In such cases, GABA can be employed as a synergistic tool alongside B fertilization to provide rapid mitigation of deficiency symptoms. To translate these findings into practical agriculture, future field trials must compare different delivery methods (e.g., foliar spray versus root drenching) and incorporate cost-effectiveness evaluations. Concurrently, evaluating GABA’s specific impact on nutrient transporter expression, gas exchange dynamics, and oxidative stress markers will further solidify the mechanistic link between GABA-mediated nutrient acquisition and overall plant performance.

## 5 Conclusion

Exogenous GABA affects the growth and mineral nutrient acquisition of trifoliate orange seedlings by maintaining nutrient homeostasis in a concentration-dependent manner. Based on a 30-day treatment, optimal GABA concentrations (0.01–0.1 mM) mitigated B-deficiency symptoms, characterized by a reconfiguration of root architecture and improved biomass production (increasing by up to 23.3%). In contrast, high GABA concentrations (0.5 and 1 mM) led to altered nutrient distribution and hindered overall seedling growth (decreasing total biomass by up to 19.2%). Collectively, this study characterizes the concentration-dependent responses of seedling growth and nutrient accumulation to exogenous GABA. Future studies should focus on elucidating the temporal dynamics and underlying molecular mechanisms (such as potential interactions with nitrogen signaling or ion channel regulation), alongside conducting long-term field trials under complex soil conditions, to translate these physiological insights into practical agricultural recommendations.

## Data Availability

The original contributions presented in the study are included in the article/supplementary material. Further inquiries can be directed to the corresponding author.
